# High-quality permanent draft genome sequence of the *Parapiptadenia rigida*-nodulating *Burkholderia* sp. strain UYPR1.413

**DOI:** 10.1186/s40793-015-0018-9

**Published:** 2015-06-04

**Authors:** Sofie E De Meyer, Elena Fabiano, Rui Tian, Peter Van Berkum, Rekha Seshadri, TBK Reddy, Victor Markowitz, Natalia Ivanova, Amrita Pati, Tanja Woyke, John Howieson, Nikos Kyrpides, Wayne Reeve

**Affiliations:** 1 Centre for Rhizobium Studies, Murdoch University, Murdoch, WA, Australia; 2 Instituto de Investigaciones Biológicas Clemente Estable, Montevideo, Uruguay; 3 Soybean Genomics and improvement laboratory Bldg 006, BARC-West USDA ARS, 10300 Baltimore Blvd, Beltsville 20705, MD, USA; 4 DOE Joint Genome Institute, Walnut Creek, CA, USA; 5 Biological Data Management and Technology Center, Lawrence Berkeley National Laboratory, Berkeley, CA, USA; 6 Department of Biological Sciences, King Abdulaziz University, Jeddah, Saudi Arabia

**Keywords:** Root-nodule bacteria, Nitrogen fixation, Rhizobia, Betaproteobacteria, GEBA-RNB

## Abstract

*Burkholderia* sp. strain UYPR1.413 is an aerobic, motile, Gram-negative, non-spore-forming rod that was isolated from a root nodule of *Parapiptadenia rigida* collected at the Angico plantation, Mandiyu, Uruguay, in December 2006. A survey of symbionts of *P. rigida* in Uruguay demonstrated that this species is nodulated predominantly by *Burkholderia* microsymbionts. Moreover, *Burkholderia* sp. strain UYPR1.413 is a highly efficient nitrogen fixing symbiont with this host. Currently, the only other sequenced isolate to fix with this host is *Cupriavidus* sp. UYPR2.512. Therefore, *Burkholderia* sp. strain UYPR1.413 was selected for sequencing on the basis of its environmental and agricultural relevance to issues in global carbon cycling, alternative energy production, and biogeochemical importance, and is part of the GEBA-RNB project. Here we describe the features of *Burkholderia* sp. strain UYPR1.413, together with sequence and annotation. The 10,373,764 bp high-quality permanent draft genome is arranged in 336 scaffolds of 342 contigs, contains 9759 protein-coding genes and 77 RNA-only encoding genes.

## Introduction

Rhizobia are soil bacteria that have acquired the ability to establish symbiotic associations with plants, mainly from the *Fabaceae* family, and carry out the Biological Nitrogen Fixation (BNF) process. BNF is catalyzed by the rhizobial nitrogenase complex, whereby N _2_ is reduced to ammonium.

Well-known and studied rhizobia are those belonging to the α-proteobacteria (eg. *Azorhizobium *, *Bradyrhizobium *, *Ensifer *, *Mesorhizobium * and *Rhizobium *). In 2001 symbiotic nitrogen fixing bacteria belonging to the group of *Betaproteobacteria * were reported as root nodule bacteria, introducing the term of Alpha and Beta-rhizobia to differentiate both groups of rhizobia [1], [2]. The Beta-rhizobia identified so far belong to only two genera: *Burkholderia * and *Cupriavidus * and the association seem to be mainly with plants from the Mimosoideae subfamily [3]. Additionally, studies indicate that the South American *Mimosa* genus is preferentially nodulated by Beta-rhizobia [4]. Different Beta-rhizobia species have been described belonging to the *Burkholderia * genus (eg. *B. caballeronis **,**B. caribensis **,** B. diazotrophica **, **B. dilworthii **,**B. mimosarum **,**B. nodosa **,**B. phymatum **,**B. rhynchosiae **, **B. sabiae **, **B. sprentiae **,**B. symbiotica * and* B. tuberum *) but only two in the *Cupriavidus * genus (*C. taiwanensis * and* C. necator *) [2], [5]–[17].

*Burkholderia * sp. UYPR1.413 strain has been isolated from a root nodule of Parapiptadenia rigida  (Benth.) Brenan found in an angico plantation in Artigas, Uruguay [18]. *P. rigida* belongs to the Mimosoideae subfamily and is a woody species, which can reach 30 m in height and a diameter of 60 to 80 cm [19]. The wood is of excellent quality, heavy, elastic, very hard and quite durable, rich in tannins and has medicinal properties [20]. There are six different species of *Parapiptadenia* in the Americas of which only *P. rigida* is present in Uruguay. A survey of symbionts of *P. rigida* in Uruguay demonstrated that this species is nodulated by rhizobia belonging to the genera *Burkholderia *, *Cupriavidus * and *Rhizobium *, of which the *Burkholderia * microsymbionts predominated [18]. *Burkholderia * sp. UYPR1.413 strain belongs to a group of microsymbionts that were able to nodulate and fix nitrogen with *P. rigida*[18]. In this work we present the description of the *Burkholderia * sp. UYPR1.413 high-quality permanent draft genome sequence and its annotation.

### Organism information

#### Classification and features

**Fig. 1:**
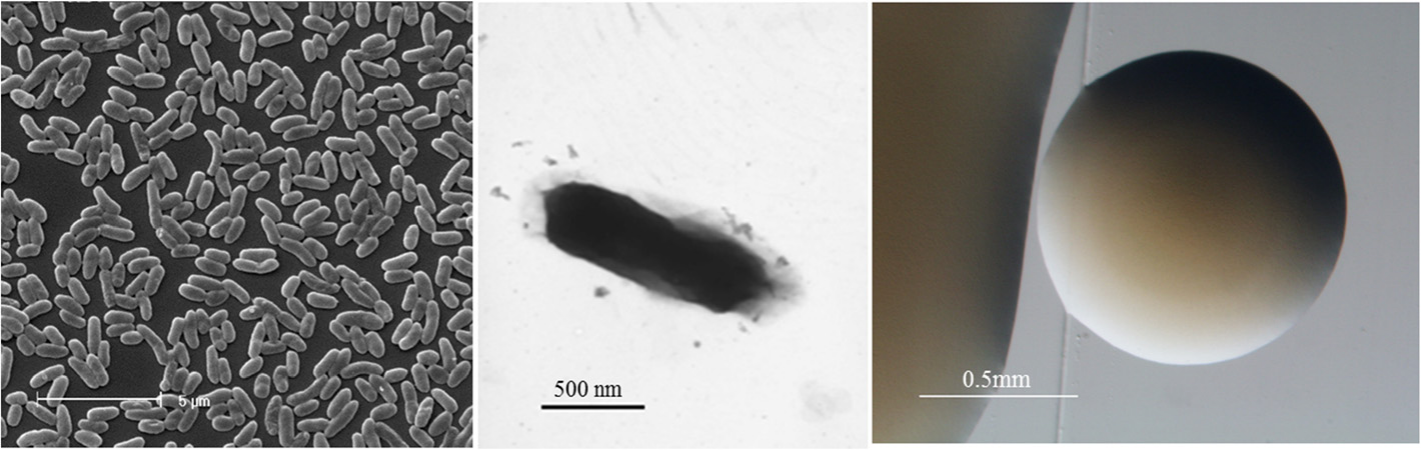
Images of *Burkholderia* sp. strain UYPR1.413 using scanning (Left) and transmission (Center) electron microscopy and the appearance of colony morphology on solid media (Right)

*Burkholderia * sp. strain UYPR1.413 is a motile, Gram-negative, non-spore-forming rod (Fig. 1 Left, Center) in the order *Burkholderiales * of the class *Betaproteobacteria *. The rod-shaped form varies in size with dimensions of 0.3–0.5 μm in width and 1.0–2.0 μm in length (Fig. 1 Left). It is fast growing, forming 0.5–1 mm diameter colonies after 24 h when grown on TY [21] at 28 °C. Colonies on TY are white-opaque, slightly domed, moderately mucoid with smooth margins (Fig. 1 Right).

**Fig. 2:**
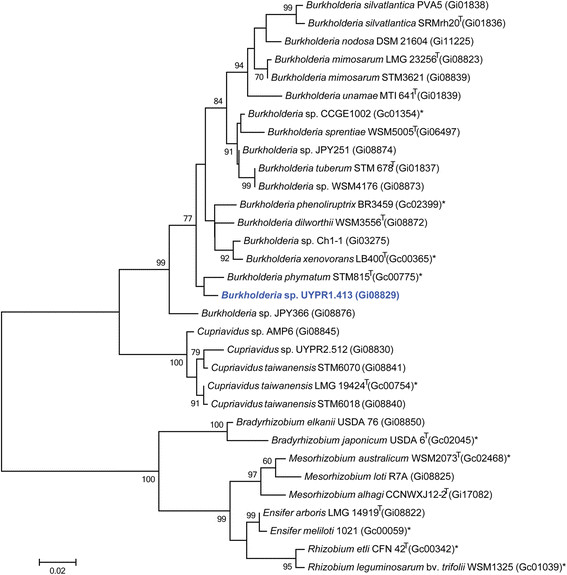
Phylogenetic tree highlighting the position of *Burkholderia* sp. strain UYPR1.413 (shown in blue print) relative to other type and non-type strains in the *Burkholderia* genus using 1046 bp internal region of the 16S rRNA gene. Several Alpha-rhizobia sequences were used as outgroup. All sites were informative and there were no gap-containing sites. Phylogenetic analyses were performed using MEGA, version 5.05 [47]. The tree was built using the maximum likelihood method with the General Time Reversible model. Bootstrap analysis with 500 replicates was performed to assess the support of the clusters. Type strains are indicated with a superscript T. Strains with a genome sequencing project registered in GOLD [30] have the GOLD ID provided after the strain number. Finished genomes are designated with an asterisk

Figure 2 shows the phylogenetic relationship of *Burkholderia * sp. strain UYPR1.413 in a 16S rRNA gene sequence based tree. This strain is phylogenetically most related to *Burkholderia sabiae * Br3407 ^T^ , *Burkholderia caribensis * MWAP64 ^T^ and *Burkholderia phymatum * STM815 ^T^ with sequence identities to UYPR1.413 16S rRNA gene sequence of 98.96, 98.64 and 98.56 %, respectively, as determined using the EzTaxon-e server [22]. *Burkholderia sabiae * Br3407 ^T^ was first isolated from root nodules of Mimosa caesalpiniifolia , a native tree to Brazil [6]. *Burkholderia caribensis * MWAP64 ^T^ was first isolated from vertisol in Martinique [5] and related strains have been identified as a plant growth promoting bacteria for grain Amaranth and Mango trees [23], [24] and nitrogen fixing root nodule bacteria for several *Mimosa* species [25], [26]. *Burkholderia phymatum * STM815 ^T^ is also known to nodulate effectively with several *Mimosa* species [27]. Minimum Information about the Genome Sequence (MIGS) [28] is provided in Table 1.

**Table 1:** Classification and general features of *Burkholderia* sp. strain UYPR1.413 in accordance with the MIGS recommendations [28] published by the Genome Standards Consortium [48]

MIGS ID	Property	Term	Evidence code
	Classification	Domain *Bacteria*	TAS [49]
		Phylum *Proteobacteria*	TAS [50], [51]
		Class *Betaproteobacteria*	TAS [52]
		Order *Burkholderiales*	TAS [53]
		Family *Burkholderiaceae*	TAS [54]
		Genus *Burkholderia*	TAS [55]
		Species *Burkholderia* sp.	IDA
		(Type) strain UYPR1.413	IDA
	Gram stain	Negative	TAS [55]
	Cell shape	Rod	IDA
	Motility	Motile	IDA
	Sporulation	non-sporulating	TAS [55]
	Temperature range	Not reported	
	Optimum temperature	28 °C	IDA
	pH range; Optimum	Not reported	
	Carbon source	Not reported	
MIGS-6	Habitat	Soil, root nodule on host	TAS [18]
MIGS-6.3	Salinity	Not reported	
MIGS-22	Oxygen requirement	Aerobic	IDA
MIGS-15	Biotic relationship	Symbiotic	TAS [18]
MIGS-14	Pathogenicity	Non-pathogenic	NAS
MIGS-4	Geographic location	Uruguay	TAS [18]
MIGS-5	Sample collection	December, 2006	TAS [18]
MIGS-4.1	Latitude	−30.507	TAS [18]
MIGS-4.2	Longitude	−57.702	TAS [18]
MIGS-4.4	Altitude	76 m	IDA

Evidence codes-IDA: Inferred from Direct Assay; TAS: Traceable Author Statement (i.e., a direct report exists in the literature); NAS: Non-traceable Author Statement (i.e., not directly observed for the living, isolated sample, but based on a generally accepted property for the species, or anecdotal evidence). These evidence codes are from the Gene Ontology project [56]

### Symbiotaxonomy

*Burkholderia * sp. strain UYPR1.413 was isolated from Parapiptadenia rigida , a Mimosoideae legume native to Uruguay [18]. This tree is native to South America, including south Brazil, Argentina, Paraguay, and Uruguay, and used by locals for timber and as a source of gums, tannins and essential oils [18]. *Burkholderia * sp. strain UYPR1.413 is able to renodulate its original host and is highly efficient in fixing nitrogen with this host [18]. A selection of host plants, including Trifolium repens *,*Medicago sativa , Peltophorum dubium  and Mimosa pudica  were investigated previously for their ability to nodulate with UYPR1.413 and only *M. pudica* plants were nodulated by UYPR1.413, albeit ineffectively [18].

### Genome sequencing information

#### Genome project history

This organism was selected for sequencing on the basis of its environmental and agricultural relevance to issues in global carbon cycling, alternative energy production, and biogeochemical importance, and is part of the Genomic Encyclopedia of Bacteria and Archaea, The Root Nodulating Bacteria chapter (GEBA-RNB) project at the U.S. Department of Energy, Joint Genome Institute (JGI) for projects of relevance to agency missions [29]. The genome project is deposited in the Genomes OnLine Database [30] and the high-quality permanent draft genome sequence in IMG [31]. Sequencing, finishing and annotation were performed by the JGI using state of the art sequencing technology [32]. A summary of the project information is shown in Table 2.

**Table 2:** Genome sequencing project information for *Burkholderia* sp. strain UYPR1.413

MIGS ID	Property	Term
MIGS-31	Finishing quality	Permanent-draft
MIGS-28	Libraries used	Illumina Std PE
MIGS-29	Sequencing platforms	Illumina HiSeq 2000
MIGS-31.2	Fold coverage	117.1 × Illumina
MIGS-30	Assemblers	Velvet version 1.1.04, ALLPATHS-LG V.r41043
MIGS-32	Gene calling methods	Prodigal 1.4
	Locus Tag	A3A7
	Genbank ID	JAFD01000000
	Genbank Date of Release	January 23, 2014
	GOLD ID	Gp0010091
	BIOPROJECT	PRJNA165303
MIGS-13	Source Material Identifier	UYPR1.413
	Project relevance	Symbiotic N _2_ fixation, agriculture

#### Growth conditions and genomic DNA preparation

*Burkholderia * sp. strain UYPR1.413 was grown to mid logarithmic phase in TY rich media [21] on a gyratory shaker at 28 °C. DNA was isolated from 60 mL of cells using a CTAB (Cetyl trimethyl ammonium bromide) bacterial genomic DNA isolation method [33].

#### Genome sequencing and assembly

The draft genome of *Burkholderia * sp. UYPR1.413 was generated at the DOE Joint genome Institute (JGI) using state of the art technology [32]. An Illumina Std shotgun library was constructed and sequenced using the Illumina HiSeq 2000 platform which generated 23,255,298 reads totaling 3488.3 Mbp. All general aspects of library construction and sequencing performed at the JGI can be found at the JGI web site [34]. All raw Illumina sequence data was passed through DUK, a filtering program developed at JGI, which removes known Illumina sequencing and library preparation artifacts (Mingkun L, Copeland A, Han J. unpublished). The following steps were then performed for assembly: (1) filtered Illumina reads were assembled using Velvet version 1.1.04 [35] (2) 1–3 Kbp simulated paired end reads were created from Velvet contigs using wgsim [36] (3) Illumina reads were assembled with simulated read pairs using Allpaths-LG (version r41043) [37]. Parameters for assembly steps were: 1) Velvet (velveth: 63-shortPaired and velvetg: –very clean yes –exportFiltered yes –min contig lgth 500 –scaffolding no –cov cutoff 10) 2) wgsim (–e 0 –1 100 –2 100 –r 0 –R 0 –X 0) 3) Allpaths-LG (PrepareAllpathsInputs: PHRED 64 = 1 PLOIDY = 1 FRAG COVERAGE = 125 JUMP COVERAGE = 25 LONG JUMP COV = 50, RunAllpathsLG: THREADS = 8 RUN = std shredpairs TARGETS = standard VAPI WARN ONLY = True OVERWRITE = True). The final draft assembly contained 342 contigs in 336 scaffolds. The total size of the genome is 10.4 Mbp and the final assembly is based on 1214.2 Mbp of Illumina data, which provides an average of 117.1× coverage of the genome.

### Genome annotation

Genes were identified using Prodigal [38], as part of the DOE-JGI genome annotation pipeline [39], [40] followed by a round of manual curation using GenePRIMP [41] for finished genomes and Draft genomes in fewer than 10 scaffolds. The predicted CDSs were translated and used to search the National Center for Biotechnology Information (NCBI) non-redundant database, UniProt, TIGRFam, Pfam, KEGG, COG, and InterPro databases. The tRNAScanSE tool [42] was used to find tRNA genes, whereas ribosomal RNA genes were found by searches against models of the ribosomal RNA genes built from SILVA [43]. Other non-coding RNAs such as the RNA components of the protein secretion complex and the RNase P were identified by searching the genome for the corresponding Rfam profiles using INFERNAL [44]. Additional gene prediction analysis and manual functional annotation was performed within the Integrated Microbial Genomes-Expert Review (IMG-ER) system [45] developed by the Joint Genome Institute, Walnut Creek, CA, USA.

### Genome properties

**Fig. 3:**
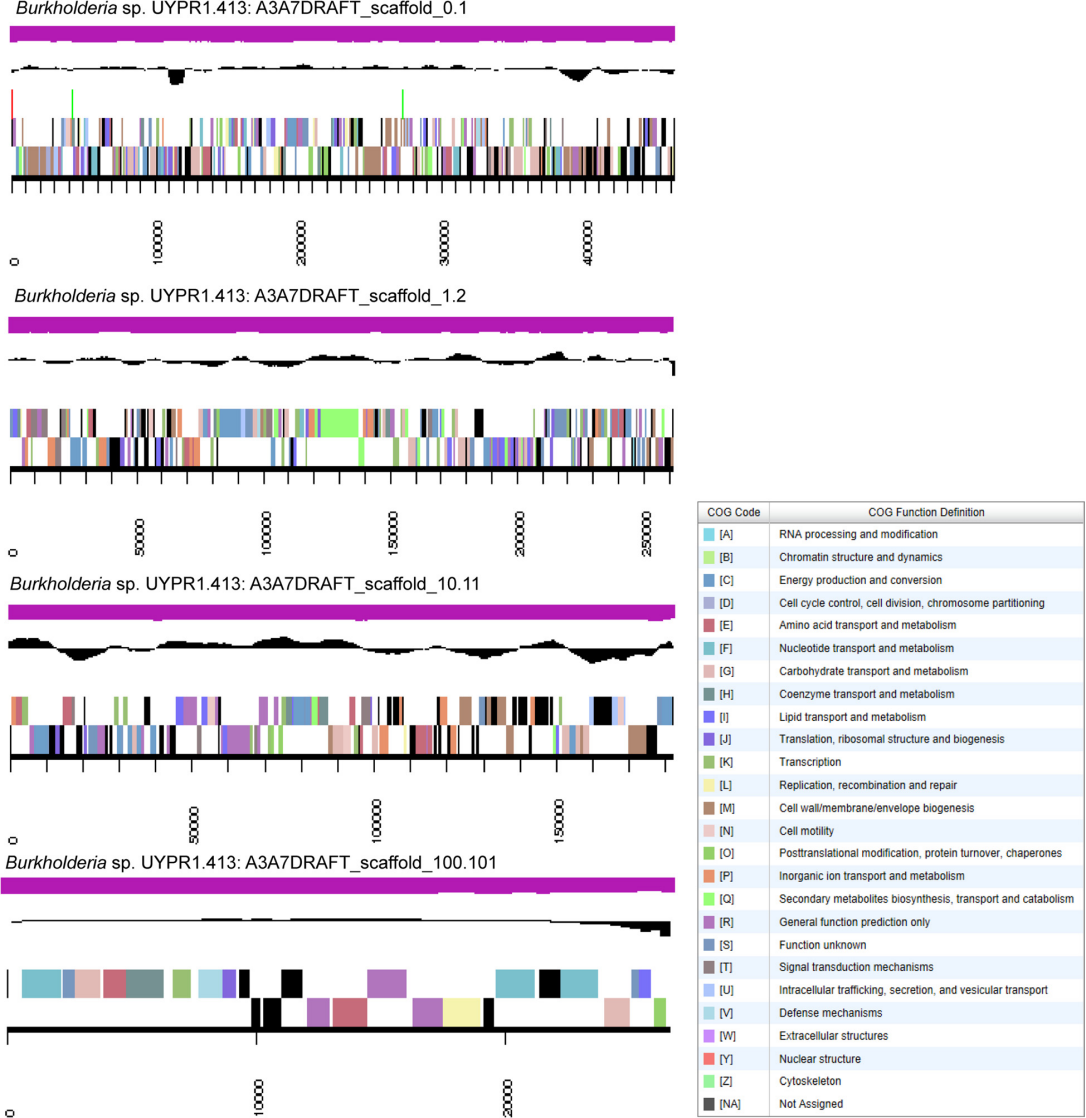
Graphical map of the four largest scaffolds of the genome of *Burkholderia* sp. strain UYPR1.413. From the bottom to the top of each scaffold: Genes on forward strand (color by COG categories as denoted by the IMG platform), Genes on reverse strand (color by COG categories), RNA genes (tRNAs green, sRNAs red, other RNAs black), GC content, GC skew

The genome is 10,373,764 nucleotides with 62.28 % GC content (Table 3) and comprised of 336 scaffolds and 342 contigs (Fig. 3). From a total of 9836 genes, 9759 were protein encoding and 77 RNA only encoding genes. The majority of genes (75.92 %) were assigned a putative function whilst the remaining genes were annotated as hypothetical. The distribution of genes into COGs functional categories is presented in Table 4.

**Table 3:** Genome statistics for *Burkholderia* sp. strain UYPR1.413

Attribute	Value	% of total
Genome size (bp)	10,373,764	100
DNA coding (bp)	8,806,315	84.89
DNA G + C (bp)	6,461,024	62.28
DNA scaffolds	336	
Total genes	9836	100
Protein-coding genes	9759	99.22
RNA genes	77	0.78
Pseudo genes	1	0.01
Genes in internal clusters	471	4.79
Genes with function prediction	7467	75.92
Genes assigned to COGs	6103	62.05
Genes with Pfam domains	7650	77.78
Genes with signal peptides	934	9.50
Genes with transmembrane helices	2097	21.32
CRISPR repeats	1	

**Table 4:** Number of protein coding genes of *Burkholderia* sp. strain UYPR1.413 associated with the general COG functional categories

Code	Value	% Age	COG Category
J	193	2.79	Translation, ribosomal structure and biogenesis
A	1	0.01	RNA processing and modification
K	721	10.42	Transcription
L	231	3.34	Replication, recombination and repair
B	4	0.06	Chromatin structure and dynamics
D	36	0.52	Cell cycle control, Cell division, chromosome partitioning
V	67	0.97	Defense mechanisms
T	332	4.80	Signal transduction mechanisms
M	405	5.85	Cell wall/membrane/envelope biogenesis
N	136	1.96	Cell motility
U	200	2.89	Intracellular trafficking, secretion, and vesicular transport
O	196	2.83	Posttranslational modification, protein turnover, chaperones
C	526	7.60	Energy production and conversion
G	527	7.61	Carbohydrate transport and metabolism
E	789	11.40	Amino acid transport and metabolism
F	103	1.49	Nucleotide transport and metabolism
H	220	3.18	Coenzyme transport and metabolism
I	325	4.70	Lipid transport and metabolism
P	308	4.45	Inorganic ion transport and metabolism
Q	248	3.58	Secondary metabolite biosynthesis, transport and catabolism
R	794	11.47	General function prediction only
S	559	8.08	Function unknown
−	3733	37.95	Not in COGS

The total is based on the total number of protein coding genes in the genome

## Conclusion

*Burkholderia * sp. UYPR1.413 belongs to a group of Beta-rhizobia isolated from Parapiptadenia rigida *,* a native tree from Uruguay belonging to the Mimosoideae legume group [18]. This tree is also native to the south of Brazil, Argentina and Paraguay [18]. Phylogenetic analysis revealed that UYPR1.413 is most closely related to *Burkholderia sabiae * Br3407 ^T^ , *Burkholderia caribensis * MWAP64 ^T^ and *Burkholderia phymatum * STM815 ^T^ . Interestingly, Br3407 ^T^ was isolated from nitrogen-fixing nodules on the roots of Mimosa caesalpiniifolia , a legume tree native to Brazil [6]. MWAP64 ^T^ has not been reported to nodulate legume plants, however *B. caribensis * TJ182 is able to nodulate and fix nitrogen with Mimosa pigra [7]. STM815 ^T^ was originally isolated from Macroptilium atropurpureum  but could not be authenticated on this host [1]. Additional studies showed that STM815 ^T^ is instead able to nodulate a wide range of *Mimosa* species [27]. Glasshouse experiments from previous studies have shown that *Burkholderia * sp. UYPR1.413 is also able to nodulate Mimosa pudica  seedlings, albeit ineffectively [18]. However, it is different from the other microsymbiont in that it can form an effective association with Parapiptadenia rigida . The only other sequenced isolate to fix with this host is *Cupriavidus * sp. UYPR2.512 [46]. There are in total 13 *Burkholderia * strains that are known legume symbionts; four (WSM3556 ^T^ , WSM4176, WSM5005 ^T^ , STM678 ^T^ ) nodulate South African papilionoid species, in contrast to the other nine (BR3459, CCGE1002, DSM 21604, JPY251, JPY366, LMG 23256 ^T^ , STM815, STM3621 and UYPR1.413) that are able to nodulate mimosoid species. A comparison of the mimosoid nodulating strains reveals that UYPR1.413 has the largest genome (10.4 Mbp), with the highest KOG count (1670) and the lowest GC (65.28 %) percentage in this group. All 13 of these genomes share the nitrogenase-RXN MetaCyc pathway catalyzed by a multiprotein nitrogenase complex. However, only *Burkholderia * sp. UYPR1.413 has been shown to fix effectively with Parapiptadenia rigida . The genome attributes of *Burkholderia * sp. UYPR1.413 will therefore be important for ongoing molecular analysis of the plant microbe interactions required for the establishment of leguminous tree symbioses with this host.

## Abbreviations

GEBA-RNB: Genomic Encyclopedia of Bacteria and Archaea-Root Nodule Bacteria

JGI: Joint Genome Institute

TY: Trypton Yeast

CTAB: Cetyl trimethyl ammonium bromide

WSM: Western Australian Soil Microbiology

BNF: Biological Nitrogen Fixation

## Competing interests

The authors declare that they have no competing interests.

## Authors’ contributions

EF supplied the strain and background information for this project, PVB supplied DNA to JGI, TR performed all imaging, SDM and WR drafted the paper, JH provided financial support and all other authors were involved in sequencing the genome and editing the final manuscript. All authors read and approved the final manuscript.
